# Gas6/Axl Axis Activation Dampens the Inflammatory Response in Osteoarthritic Fibroblast-like Synoviocytes and Synovial Explants

**DOI:** 10.3390/ph16050703

**Published:** 2023-05-06

**Authors:** Juliana P. Vago, Natália Valdrighi, Esmeralda N. Blaney-Davidson, Daniel L. A. H. Hornikx, Margot Neefjes, María E. Barba-Sarasua, Nathalie G. M. Thielen, Martijn H. J. van den Bosch, Peter M. van der Kraan, Marije I. Koenders, Flávio A. Amaral, Fons A. J. van de Loo

**Affiliations:** 1 Experimental Rheumatology, Department of Rheumatology, Radboud Institute for Molecular Life Sciences, Radboud University Medical Center, 6525 GA Nijmegen, The Netherlands; 2 Departament of Biochemistry and Immunology, Institute of Biological Sciences, Universidade Federal de Minas Gerais, Belo Horizonte 31270-901, Minas Gerais, Brazil

**Keywords:** osteoarthritis, TAM receptors, Gas6, synovitis

## Abstract

Osteoarthritis (OA) is the most prevalent joint disease, and it is characterized by cartilage degeneration, synovitis, and bone sclerosis, resulting in swelling, stiffness, and joint pain. TAM receptors (Tyro3, Axl, and Mer) play an important role in regulating immune responses, clearing apoptotic cells, and promoting tissue repair. Here, we investigated the anti-inflammatory effects of a TAM receptor ligand, i.e., growth arrest-specific gene 6 (Gas6), in synovial fibroblasts from OA patients. TAM receptor expression was determined in synovial tissue. Soluble Axl (sAxl), a decoy receptor for the ligand Gas6, showed concentrations 4.6 times higher than Gas6 in synovial fluid of OA patients. In OA fibroblast-like synoviocytes (OAFLS) exposed to inflammatory stimuli, the levels of sAxl in the supernatants were increased, while the expression of Gas6 was downregulated. In OAFLS under TLR4 stimulation by LPS (*Escherichia coli* lipopolysaccharide), the addition of exogenous Gas6 by Gas6-conditioned medium (Gas6-CM) reduced pro-inflammatory markers including IL-6, TNF-α, IL-1β, CCL2, and CXCL8. Moreover, Gas6-CM downregulated IL-6, CCL2, and IL-1β in LPS-stimulated OA synovial explants. Pharmacological inhibition of TAM receptors by a pan inhibitor (RU301) or by a selective Axl inhibitor (RU428) similarly abrogated Gas6-CM anti-inflammatory effects. Mechanistically, Gas6 effects were dependent on Axl activation, determined by Axl, STAT1, and STAT3 phosphorylation, and by the downstream induction of the suppressors of the cytokine signaling family (SOCS1 and SOCS3). Taken together, our results showed that Gas6 treatment dampens inflammatory markers of OAFLS and synovial explants derived from OA patients associated with SOCS1/3 production.

## 1. Introduction

Osteoarthritis (OA) is a musculoskeletal disorder and the most prevalent form of arthritis, considered the leading cause of joint pain and disability. OA is a multifactorial disease in which genetic and environmental factors contribute to its development and progression. The major locations affected are the hand, shoulder, hip, and the knee joints, which are most prevalently affected. Pathologies like degradation of the articular cartilage, subchondral bone sclerosis, and synovial inflammation develop along the progression of OA and are accompanied by severe joint pain [1]. Symptoms are typically managed through a combination of non-pharmacological procedures and the administration of anti-inflammatory drugs focused on pain management. For end-stage disease, surgical joint replacement is the only treatment option available.

The pathogenesis of OA is marked by tissue damage accumulated over time, which locally triggers an innate immune reaction [2]. This synovial inflammation (synovitis) supports a significant contribution to OA progression [3,4]. The inflammatory process is maintained by damage-associated molecular patterns (DAMPs), e.g., S100A8/A9, derived from cartilage and the inflamed synovium, which activate TLR4. Thus, this effect induces the production of cytokines (e.g., IL-6, IL-1β, and TNF) and chemokines (e.g., CXCL8, CCL2, and CCL5), which favor a proinflammatory environment in the joints of OA patients promoting a positive feedback loop [5,6,7]. The triggered inflammatory pathways upregulate several catabolic mediators and cartilage matrix-degrading proteases in the joints, which might cause more severe pain and tissue injury [8]. 

TAM receptors (Tyro3, Axl, and Mer) play a critical role in modulating the immune system and tissue homeostasis, via efferocytosis (phagocytosis of apoptotic cells) [9]. The efferocytosis is mediated by TAM receptor ligands, namely growth arrest-specific 6 (Gas6) and protein S (Pros1), which facilitate the interaction between TAM receptors in phagocytes and phosphatidylserine in apoptotic cells [9,10]. Gas6 interacts with all three receptors, exhibiting the highest affinity for Axl, while Pros1 interacts only with Tyro3 and Mer [10]. Gas6 has been described to inhibit the production of pro-inflammatory cytokines in vitro (TNF, IL-6, and IL-1β) [11]. In the context of inflammatory diseases, activation of TAM receptors by Gas6 has been associated with downregulation of inflammatory cytokines [11,12,13] and induction of pro-resolving mediators contributing to inflammation resolution [14].

Emerging evidence suggests that TAM receptors might play a significant and multifaceted role in arthritis [15]. TAM receptors and Gas6 have been described as being expressed in synovial tissue of rheumatoid arthritis (RA) patients [16,17], in which Gas6 levels were lower in erosive RA compared with non-erosive RA [18]. In addition, the soluble form of Axl, which acts as a decoy receptor and may reduce the interaction of Gas6 with membrane-bound receptors [19,20], was detected in the synovial fluids of RA and OA patients [16,21,22]. We have previously shown that overexpression of the TAM receptor ligand genes *Pros1* or *Gas6* successfully reduced arthritis pathology in a murine model of collagen-induced arthritis [23]. Moreover, in the context of TAM deficiency, we described that TAM receptors play a protective role in mouse models of RA [24,25,26]. Although the role of TAM receptors has been more widely studied for RA, their involvement in the pathophysiology of OA has not yet been elucidated. Thus, in this study, we investigated the effect of Gas6 exposure on the inflammatory response of synovial fibroblasts obtained from patients with osteoarthritis.

## 2. Results

### 2.1. TAM Receptors Are Distinctively Expressed in OA Synovial Tissue

**Figure 1:**
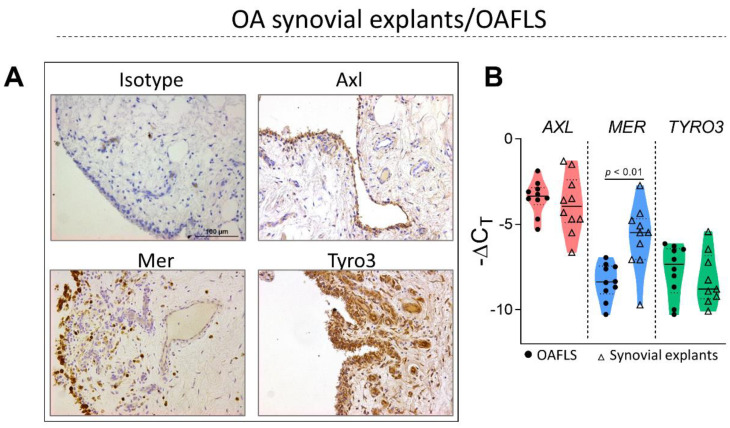
Determination of TAM receptor expression in synovial tissue of osteoarthritis patients. Synovial biopsies (*n* = 8) from osteoarthritis (OA) patients were processed for immunohistochemical staining of Axl, Mer, Tyro3, and IgG isotype control (**A**). Sections were counterstained with hematoxylin. Pictures were taken at 20× magnification. Expression of *AXL*, *MER,* and *TYRO3* in synovial explants freshly isolated from individual patients (*n* = 10), and OA synovial fibroblasts (OAFLS) (*n* = 10) were determined by qPCR (**B**). Results are shown as the mean ± SEM; *p* values were determined by unpaired Student’s *t*-test.

TAM receptor expression was determined by immunohistochemistry on the OA synovium tissue (Figure 1A). Tyro3-positive cells were identified mainly as lining cells, as well as blood vessels, particularly endothelial cells. Mer-positive cells were observed in both the lining and sublining compartments. Axl-positive cells were only detected in synovial lining cells (Figure 1A). The gene expression of TAM receptors was determined in OA synovium (synovial explants freshly isolated) and OA synovial fibroblasts (OAFLS, passages 4 to 8). The expression of *TYRO3* and *AXL* was similar among synovium and OAFLS, while the expression of *MER* was reduced in OAFLS when compared with synovial explants (Figure 1B). These results showed that TAM receptors can be expressed in different compartments of the OA synovium, but all TAM receptors were detected in the lining cells.

### 2.2. The Soluble Forms of TAM Receptors Are Present in OA Synovial Fluid with Higher Levels of sAxl

**Figure 2:**
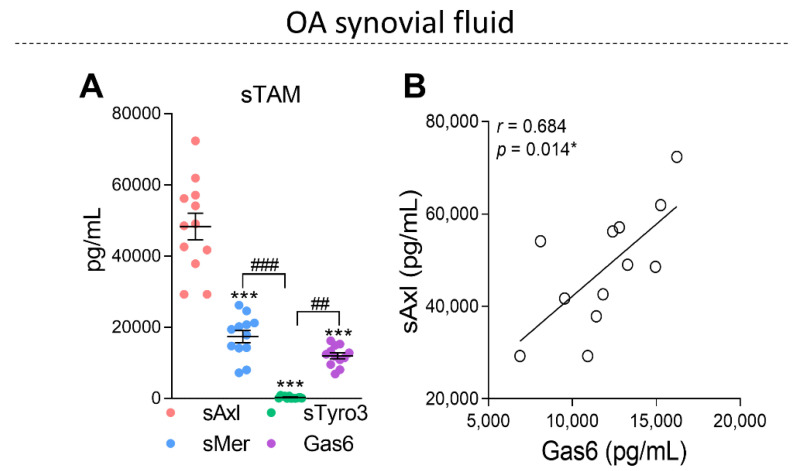
Determination of soluble TAM receptors and Gas6 levels in synovial fluid of osteoarthritis patients. Soluble Axl (sAxl), soluble Mer (sMer), soluble Tyro3 (sTyro3), and Gas6 levels were determined in synovial fluids of OA patients (*n* = 12) by ELISA (**A**). The correlation between sAxl and Gas6 in synovial fluid was evaluated by Pearson’s coefficients (**B**). Results are shown as the mean ± SEM. * *p* < 0.05 and *** *p* < 0.001 when comparing sAxl with sMer, sTyro3, and Gas6. ^##^ *p* < 0.01 and ^###^ *p* < 0.001 when comparing sMer versus sTyro3, and sTyro3 versus Gas6, respectively; *p* values were determined by ANOVA with post hoc Tukey’s test (multiple groups).

It has been shown that TAM receptors are cleaved in an inflammatory milieu, generating soluble forms [19,27]. In this regard, the soluble forms of TAM receptors (sTAM) and Gas6 were evaluated in the synovial fluid of OA patients (OASF). Interestingly, the levels of sAxl were predominant in the OASF when compared with sMer and sTyro3 (Figure 2A). The amount of sAxl found was ~4.6 times higher than the amount of Gas6 (Figure 2A). A positive correlation between sAxl and Gas6 was observed in OASF (Figure 2B) without correlations among Gas6 and sMer or sTyro3 (Supplementary Figure S1). Because Gas6 has been suggested to exhibit anti-inflammatory effects [11,28], the correlations between the soluble forms of TAM receptors, Gas6, and inflammatory cytokines were also investigated in OASF (Table 1). No correlations were observed when comparing Gas6 and inflammatory cytokines or when comparing TAM-soluble forms and inflammatory cytokines; however, a trend for a positive correlation between sAxl and TNF-α was observed. The lack of correlation among Gas6 versus inflammatory cytokines in association with the increased levels of sAxl in the synovial fluids suggests that sAxl may act as a decoy receptor in the OA joints and may be responsible for inhibiting the Gas6-mediated anti-inflammatory effects.

### 2.3. sAxl Is Increased in OAFLS Supernatants under Inflammatory Stimuli

**Figure 3:**
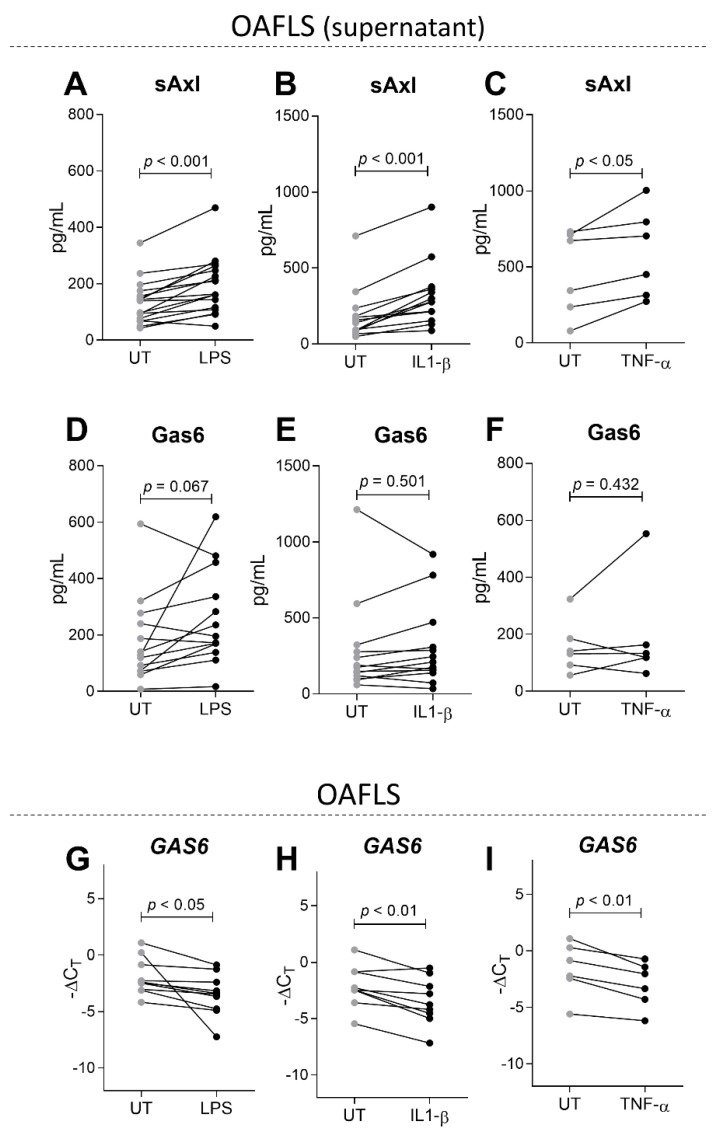
sAxl and Gas6 levels in OAFLS supernatants and *GAS6* expression in OAFLS under an inflammatory milieu. OA synovial fibroblasts (OAFLS) were stimulated with LPS (10 ng/mL) or the recombinants human IL-1β (0.1 ng/mL) and TNF-α (1 ng/mL) for 24 h. Soluble Axl (sAxl) (**A**–**C**) and Gas6 (**D**–**F**) levels were determined by ELISA. *GAS6* expression in OAFLS was determined by qPCR (**G**–**I**). Results are shown as the mean ± SEM; *p* values are shown in the figure and were determined by paired Student’s *t*-test.

To assess the influence of inflammation on sAxl and Gas6 levels in the context of osteoarthritis in vitro, OAFLS were stimulated with LPS (10 ng/mL) and the recombinants of human IL-1β (0.1 ng/mL) or TNF-α (1 ng/mL) for 24 h. A dose response for these stimuli and the expression of inflammatory markers were determined (Supplementary Figure S2). In an inflammatory milieu, sAxl levels were increased in the supernatants of OAFLS, regardless of the type of stimulus (Figure 3A–C) while Gas6 levels in the supernatants were not changed at 24 h post-stimulus (Figure 3D–F). No correlations between sAxl and Gas6 in the supernatants of unstimulated or stimulated OAFLS were observed. It was already described that Gas6 expression is downregulated in macrophages under a proinflammatory environment [29]; thus *GAS6* expression was also evaluated in our experimental settings. Interestingly, *GAS6* expression was reduced in OAFLS after stimulation with LPS, IL-1β, or TNF-α (Figure 3G–I). The increased sAxl levels in the supernatants of OAFLS and in OA synovial fluids (Figure 2) suggest that to be effective in dampening OA inflammation, Gas6 levels must be increased to overcome the scavenging potential of sAxl.

### 2.4. Activation of TAM Receptors by Gas6 Downregulates Pro-Inflammatory Markers in OAFLS and OA Synovial Explants

**Figure 4:**
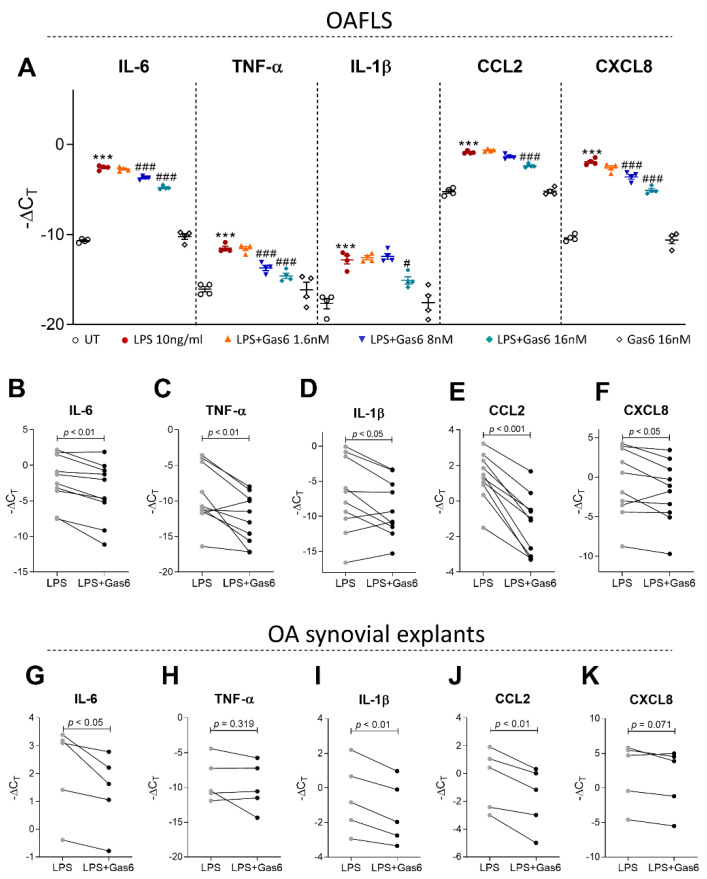
Effect of Gas6-CM on inflammatory OAFLS cytokine production. OA synovial fibroblasts (OAFLS) were pre-treated with Gas6-CM (16, 8, and 1.6 nM) for 1 h and then stimulated for 24 h with LPS (10 ng/mL). Cells were processed for qPCR analyses of the pro-inflammatory cytokines IL-6, TNF-α, IL-1β, CCL2, and CXCL8 (**A**). OAFLS (*n* = 10) and synovial explants (*n* = 5) were pre-treated with Gas6-CM (16 nM) for 1 h and then stimulated for 24 h with LPS (10 ng/mL). Cells were processed for qPCR analyses of the pro-inflammatory cytokines IL-6 (**B**,**G**), TNF-α (**C**,**H**), IL-1β (**D**,**I**), CCL2 (**E,J**), and CXCL8 (**F**,**K**). Results are shown as the mean ± SEM. *** *p* < 0.001 when comparing LPS with untreated (UT) group. ^#^ *p* < 0.05 and ^###^ *p* < 0.001 when comparing Gas6 treated with LPS group; *p* values were determined by ANOVA with post hoc Tukey’s test (multiple groups) and paired Student’s *t*-test (when comparing 2 groups).

Next, experiments were performed using a Gas6-conditioned medium (Gas6-CM) [30,31,32,33]. The efficacy of the Gas6-CM was determined by an efferocytosis assay co-culturing THP-1 cells with apoptotic neutrophils. Gas6-CM promoted the efferocytosis of apoptotic neutrophils, which was abrogated by a pan-TAMR inhibitor (RU301) (Supplementary Figure S3). To examine whether Gas6 can have protective effects on OA synovitis, OAFLS under LPS stimulation were concentration-dependently treated with Gas6-CM (Figure 4A). Gas6-CM reduced the expression of the inflammatory markers evaluated (Figure 4A), and a concentration of 16 nM was the most effective. IL-6 and CCL2 protein levels were also reduced in the OAFLS supernatants after Gas6-CM treatment (Supplementary Figure S4) without changes in CXCL8. The levels of TNF-α and IL-1β in the supernatants were below the standard curve detection level. Moreover, Gas6-CM treatment reduced the inflammatory markers IL-6, TNF-α, IL-1β, CCL2, and CXCL-8 (Figure 4B–F) in OAFLS from different patients. To better simulate OA inflammation pathology in humans, the effect of Gas6-CM was also evaluated in OA synovial explants. Gas6-CM treatment reduced IL-6, IL-1β, and CCL2 of LPS-stimulated synovial explants (Figure 4G,I,J), and a trend for CXCL8 reduction was observed (Figure 4K). Although a reduction in TNF-α for some patients was observed, this effect was not statistically significant when considering all patients (Figure 4H). These results suggest that exogenous Gas6 can be effective in reducing inflammatory mediators associated with OA pathology in OAFLS. 

### 2.5. Gas6 Anti-Inflammatory Effects Are Dependent on Axl Receptor

**Figure 5:**
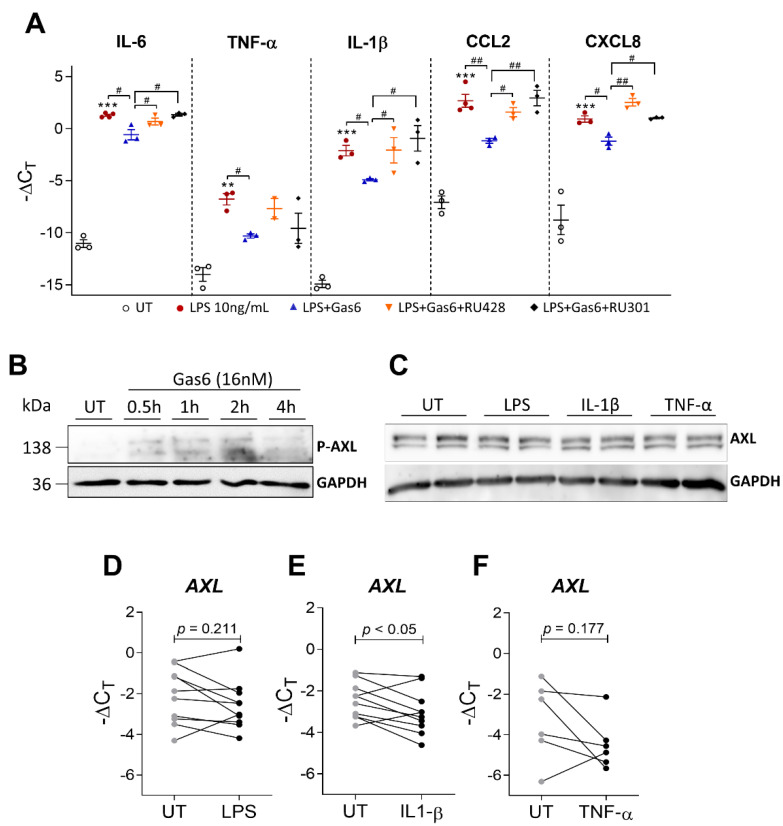
Pharmacological effects of TAM inhibition in OAFLS, and determination of Axl phosphorylation and Axl expression. OAFLS were pre-treated with a pan-TAM inhibitor RU301 (10 μM), or a selective Axl inhibitor RU428 (1 μM) for 1 h, followed by Gas6-CM (16 nM) for an additional 1 h and then were stimulated for 24 h with LPS (10 ng/mL). Cells were processed for qPCR analyses of the pro-inflammatory cytokines IL-6, TNF-α, IL-1β, CCL2, and CXCL8 (**A**). OA synovial fibroblasts (OAFLS) were treated with Gas6-CM (16 nM) for different time points and processed for Western blotting analyses for Axl phosphorylation (**B**). OA synovial fibroblasts (OAFLS) were stimulated with LPS (10 ng/mL) and the recombinants human IL-1β (0.1 ng/mL) and TNF-α (1 ng/mL) for 24 h. Axl expression in OAFLS was determined by Western blotting analysis (**C**) and qPCR (**D**–**F**). For loading control, membranes were reprobed with anti-GAPDH. Results are shown as the mean ± SEM. ** *p* < 0.01 and *** *p* < 0.001 when comparing with untreated (UT) group. ^#^ *p* < 0.05, and ^##^ *p* < 0.01 when comparing with LPS + Gas6 treated group; *p* values were determined by ANOVA with post hoc Tukey’s test (multiple groups) or paired Student’s *t*-test (when comparing 2 groups).

We next examined whether Gas6 anti-inflammatory effects were dependent on TAM receptor activation. For this purpose, TAM receptors and Axl receptor alone were inhibited by a pan-TAM receptor inhibitor (RU301) and a selective Axl inhibitor (RU428), respectively, prior to Gas6-CM treatment of LPS-stimulated OAFLS. Interestingly, the anti-inflammatory effects of Gas6-CM downregulating IL-6, IL-1β, CCL2, and CXCL8 were abrogated by both RU301 and RU428 to the same magnitude (Figure 5A), suggesting that Axl is the main receptor responsible for mediating Gas6 anti-inflammatory effects in OAFLS. In fact, Gas6-CM induced the phosphorylation of Axl receptor (Figure 5B) in OAFLS. P-Axl was detected from 30 min to 2 h after Gas6 treatment. As increased sAxl levels were observed in OAFLS supernatants under an inflammatory milieu (Figure 3), the protein amount of the Axl receptor in cells was also evaluated after pro-inflammatory stimuli. Interestingly, the protein levels of Axl in OAFLS were not changed after stimulation with LPS, IL-1β, or TNF-α (Figure 5C). These observations were also confirmed by qPCR for LPS and TNF-α stimuli, where the expression of Axl did not change (Figure 5D,F). However, downregulation of Axl was observed after IL-1β stimulation (Figure 5E). These results suggest that Gas6 mediates its anti-inflammatory effects through the Axl receptor.

### 2.6. Gas6 Induces STAT-SOCS Signaling in OAFLS

**Figure 6:**
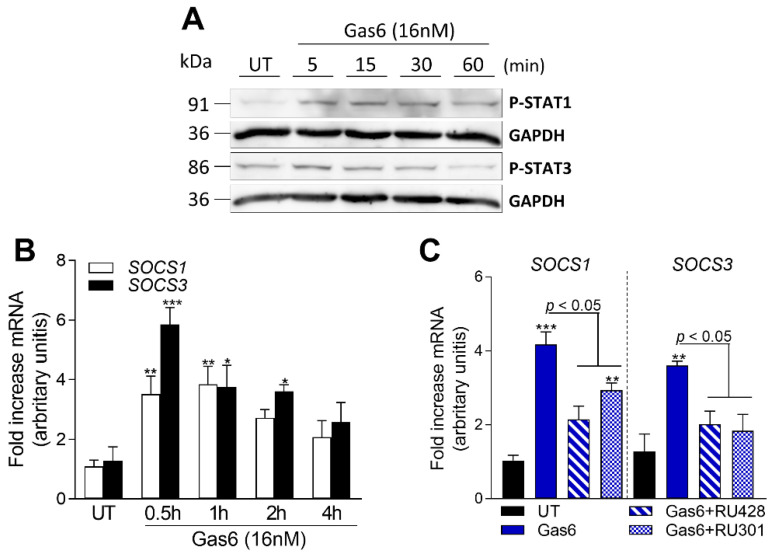
Evaluation of signaling pathways induced by Gas6 in OAFLS. OA synovial fibroblasts (OAFLS) were treated with Gas6-CM (16 nM) for different time points. Cells were processed for Western blotting analyses and the levels of P-STAT1 and P-STAT3 (**A**) were determined. For loading control, membranes were reprobed with anti-GAPDH. Cells were also processed for qPCR analyses of the suppressors of the cytokine signaling family 1/3 (*SOCS1/3*) (**B**). Results are shown as the mean ± SEM. * *p* < 0.05, ** *p* < 0.01, and *** *p* < 0.001 when comparing Gas6-treated with untreated (UT) group; *p* values were determined by ANOVA with post hoc Tukey’s multiple comparison test. OAFLS were pre-treated with a pan-TAM inhibitor RU301 (10 μM) or a selective Axl inhibitor RU428 (1 μM) for 1 h, followed by Gas6-CM (16 nM) for an additional 1 h. Cells were processed for qPCR analyses of *SOCS1/3* (**C**). Results are shown as the mean ± SEM. ** *p* < 0.01 and *** *p* < 0.001 when comparing with untreated (UT) group; *p* values were determined by ANOVA with post hoc Tukey’s test (multiple groups).

Previous studies have demonstrated that TAM receptor activation is associated with upregulation of the suppressors of the cytokine signaling family 1/3 (SOCS1/3) [12,23,34,35], well-known molecules that negatively regulate the JAK-STAT cascade [36]. To further address by which mechanism Gas6 exerts its anti-inflammatory effects on OAFLS, Axl downstream signaling pathways were determined. STAT3 and STAT1 phosphorylation were observed after Gas6-CM treatment of OAFLS (Figure 6A). Moreover, increased expression of SOCS1 and SOCS3 was also detected (Figure 6B) after 30 min of Gas6-CM treatment. We next examined whether SOCS1/3 expression was dependent on Axl activation by blocking Gas6 interaction with TAM receptors by RU301 and Axl by RU428 pre-treatment of cells. Interestingly, both RU301 and RU428 similarly inhibited Gas6-CM-induced SOCS1/3 (Figure 6C). Taken together, these results suggest that the Gas6/Axl axis anti-inflammatory effects observed were paralleled with the induction of SOCS1/3 expression.

## 3. Discussion

In this study, we examined the expression of Gas6 and TAM receptors in the synovial tissue of OA patients and the effect of exogenous Gas6 exposure on the inflammatory response of synovial fibroblasts in the context of OA. Our findings demonstrated that TAM receptors are expressed in OA synovial tissue and detected in synovial fluids in their soluble forms, and that the activation of Axl receptor in OAFLS and synovial explants by Gas6 can reduce the expression of pro-inflammatory cytokines, most likely in a SOCS1/3-dependent mechanism.

TAM receptors mediate negative feedback signaling in the immune system, controlling the inflammation process. In this sense, the role of TAM receptors in the joints seems to be relevant, as mice lacking TAM receptors (triple knockout) develop bone marrow edema, a pre-stage (pre-clinical phase) of arthritis [25]. Here, we determined the expression of TAM receptors in the synovium of OA patients. The expression of Axl has been described in the joints of RA patients [17] as well as in murine models of arthritis [24,37]. More specifically, it has been described that Axl is highly expressed by a distinct subset of CX_3_CR1^+^ tissue-resident macrophages, which form an internal immunological barrier at the synovial lining [37]. Here we demonstrated that Axl is also expressed in OAFLS and synovial explants in the same order of magnitude, and that Axl was detected predominantly in the lining compartment of the synovium. Also, our data showed higher expression of Mer in synovial explants from OA patients when compared to OAFLS, which we speculate is due to the presence of macrophage subtypes in the OA synovium. Recent studies using single-cell RNA sequencing analyses of synovial tissue have identified distinct subsets of synovial macrophages expressing Mer [38,39]. In RA patients, a specific anti-inflammatory subset of macrophages displayed upregulation of Mer [38]. More recently, synovial macrophages from RA patients revealed two subpopulations (MerTK^+^TREM2^+^ and MerTK^+^LYVE1^+^) with unique remission transcriptomic signatures enriched in negative regulators of inflammation [39]. The authors concluded that sustained RA remission is actively maintained by MerTK^+^ macrophages. 

Shedding of TAM receptors and generation of soluble forms can reduce TAM-mediated anti-inflammatory signaling by decreasing the amount of membrane-bound TAM receptors and/or by acting as a decoy receptor, capturing TAM receptor ligands in the extracellular compartment [19,20,27,40,41]. Either way, TAM activation on the cell surface can be impaired. In OA synovial fluids, the correlations between Gas6 and sAxl were positive, and the levels of sAxl were 4.6 times higher than Gas6. In fact, among the soluble forms of TAM receptor, Gas6 has the highest affinity for sAxl [32]. A limitation of our study is that no healthy donor controls were included in the analysis of synovial fluid. It has already been demonstrated in serum and plasma of healthy donors that all Gas6 in circulation is bound to sAxl [20]. Increased levels of sAxl have also been observed in several diseases [21,42,43,44,45,46,47], suggesting that the amount of Gas6 available (unbound to sAxl) may be insufficient to stimulate the intact TAM receptors on the cell surface in this milieu. Increased levels of sAxl have been described as being elevated in the serum of OA patients compared to healthy donors [21]. This effect was directly related to the severity of OA as determined by radiographic analyses. Gas6 levels were also increased in OA patients and showed a positive correlation with sAxl. Although these analyses were carried out in serum, they corroborate our data in synovial fluid, which suggests that TAM receptor activation may be compromised in the context of OA.

Murine peritoneal macrophages under LPS stimulation displayed Gas6 downregulation [29]. Deng et al. showed that Gas6 downregulation is dependent on NF-κB activation [11]. In the context of arthritis, conflicting findings regarding the levels of Gas6 and its role in the RA pathophysiology have been reported [18,21,39,48]. Reduced plasma levels of Gas6 were described in RA patients when compared with healthy control subjects [18]. Furthermore, decreased Gas6 levels were found in RA patients with erosive disease when compared with nonerosive disease [18]. In contrast, increased levels of circulating Gas6 were detected after induction of arthritis by a K/BxN serum-transfer model in mice [48]. Interestingly, plasma levels of Gas6 in RA patients have been reported to positively correlate with disease activity scores (DAS-28), erythrocyte sedimentation rate (ESR), leukocytosis, and IL-6 [18]. In addition, increased serum levels of Gas6 were described in OA patients when compared with healthy controls [21]. Based on these studies in mice and humans, we speculate that changes in Gas6 expression in arthritic diseases may be due to a compensatory mechanism in an attempt to control inflammation. As recently reported, Gas6 is expressed in a specific sublining layer cluster of FLS (THY^+^CXCL14^+^), and its expression was increased in FLS of patients in disease remission compared to active RA [39].

TAM receptor activation by Gas6 and Pros1 has been suggested to induce anti-inflammatory effects mediated by upregulation of SOCS1 and SOCS3 [12,23,34,35]. In a model of collagen-induced arthritis, our group demonstrated that overexpression of Gas6 or Pros1 in the joint cavity was protective and decreased proinflammatory cytokine production in the knee joints, increasing the expression of SOCS1/3 [23]. Gas6 overexpression was accompanied by changes in histopathological scores, with reductions in many parameters including knee inflammation, knee swelling, cartilage degradation, and bone erosion [23]. Indeed, proinflammatory cytokines have been implicated in arthritis pathogenesis [49]. In OA, inflammatory cytokines influence the balance of cartilage matrix degeneration and repair, leading to excessive production of articular proteolytic enzymes responsible for cartilage breakdown, which in turn amplifies synovial inflammation, creating a vicious circle [3,50]. The cytokines evaluated in this study were chosen based on findings that demonstrated their importance for the pathogenesis of OA, especially their impact on synovial tissue and the development of synovitis [7]. The ability of TAM receptors to restrain the overproduction of inflammatory cytokines is particularly important in the context of OA, as these cytokines can sustain the inflammatory process. For instance, CCL2, a chemoattractant for monocytes and macrophages, has been associated with OA severity and symptoms such as pain, by inducing recruitment and accumulation of activated macrophages in the joints [51]. Indeed, higher levels of CCL2 in the peripheral blood of OA patients when compared to healthy donors have been reported [52].

Pharmacological strategies on targeting inflammatory cytokines well-known to be involved in OA pathogenesis, including TNF-α and IL-1β, have been conducted [53,54]. However, some studies have shown that blocking these cytokines individually has failed to prevent the progression of OA [53,55]. It is important to consider that OA pathogenesis is not dependent only on a single cytokine, since downstream signaling pathways associated with the onset and progression of the disease can be activated by different mediators. Here, we showed no correlation between Gas6 and inflammatory cytokine levels in the synovial fluids of OA patients associated with increased levels of sAxl, suggesting that sAxl may act as a decoy receptor inhibiting Gas6 effects in the OA joints. Importantly, despite the increased shedding of Axl observed in OAFLS supernatants under inflammatory stimuli (Figure 3), we showed that the amount of intact Axl on cells (Figure 1 and Figure 5C) was sufficient to mediate the Gas6 anti-inflammatory signal. Thus, we demonstrated that the activation of the Gas6/Axl axis is essential in reducing important inflammatory cytokines associated with OA pathogenesis, including IL-6, CCL2, TNF-α, IL-1β, and CXCL8, in OAFLS and OA synovial explants. 

In summary, the present study described for the first time that Gas6 displayed anti-inflammatory effects in cells and biopsies derived from the joints of OA patients and that this effect was associated with SOCS1/3 upregulation mediated by Gas6/Axl receptor interaction. Thus, we propose that Gas6-based treatment of OA patients might be efficacious in reducing inflammation and possibly OA-related pain, as Gas6 had the strongest effect on CCL2 and IL-6.

## 4. Materials and Methods

### 4.1. Patient Material

A total of 32 patients were included in this study. The procedures were in accordance with the Helsinki Declaration of 1964, as revised in 2013. OA knee synovial explants (*n* = 10; 6 female, 4 male; mean age, 72 years; range, 56 to 83 years) and synovial fibroblasts (OAFLS) (*n* = 10; 6 female, 4 male; mean age, 68 years; range, 56 to 81 years) were isolated from patients undergoing total knee arthroplasty (TKA) at the department of Orthopedics (Radboud University Medical Center) and the Sint Maartenskliniek (Nijmegen, The Netherlands). The Kellgren–Lawrence radiographic changes were grade 3 or 4. A TKA had been recommended to all patients. Samples were obtained with informed consent prior to surgery in an anonymized manner. The synovial fluids were taken from OA patients (*n* = 12; 7 female, 5 male; mean age, 57 years; range, 41 to 67 years) during appointments at the polyclinic to alleviate pressure and pain in knee joints due to edema or swelling (synovial inflammation). Written informed consent was obtained from all patients.

### 4.2. OA Fibroblast-like Synoviocyte and Synovial Explant Isolation

Synovial samples were selected by visual inspection, separating fat, cartilage, and bone tissue from synovial tissue. Biopsy-derived synovial tissue was digested with Liberase TM (50 µg/mL) (Roche Diagnostics, Basel, Switzerland) for 1 h at 37 °C in RPMI medium (Gibco). OAFLS were cultured in RPMI enriched with 10% heat-inactivated fetal calf serum (FCS), pyruvate (1 mM), and 1% penicillin/streptomycin. Cells were maintained at 37 °C and 5% CO_2_. The medium was refreshed weekly. Cells between passages 4 and 8 were used for experiments. Synovial explants were obtained by punched biopsies (3 mm) from synovial tissue and were incubated at 37 °C and 5% CO_2_ overnight. 

### 4.3. Synovial Fluid Preparation

The synovial fluids were centrifuged at 1700× *g* for 10 min at 4 °C, followed by 30 min at 10,000× *g* at 4 °C to remove cells and debris. Supernatants were aliquoted and stored at −80 °C. Synovial fluid samples were thawed and treated with 75 U/mL of hyaluronidase (H3506; Sigma-Aldrich, Saint Louis, MO, USA) for 15 min at 37 °C to reduce viscosity and, subsequently, centrifuged at 1000× *g* for 10 min at 4 °C. Samples were aliquoted and stored at −20 °C until further analysis.

### 4.4. Cloning and Lentivirus Production for Gas6 Overexpression

For lentiviral vector production, the third-generation self-inactivating (SIN) lentiviral vector system was used. Vectors for human *GAS6* (pLV[Exp]-EGFP:T2A:Puro-EF1A > hGAS6) (VectorBuilder, Guangzhou, China) were in *Escherichia coli* hosts. Plasmids were purified from bacterial culture using Maxiprep kits (Qiagen). Viral supernatants were generated in Lenti-X 293T cells (TakaraBio, Kusatsu, Shiga, Japan) using 1 mg/mL polyethylenimine (PEI; Polysciences, Warrington, PA, USA), 5.3 µg of plasmid DNA vector, 4.0 µg MDL packaging plasmid, 3.5 µg of vesicular stomatitis virus glycoprotein G (VSV-G) envelope expression plasmid, and 1.8 µg of RSV-REV packaging expression plasmid. Viral supernatant was concentrated using Lenti-X concentrator (TakaraBio) according to the manufacturer’s protocol. Lentiviral concentration was determined using a p24 INNOTEST ELISA assay (Fujirebio, Ghent, Belgium). Cells were transduced for 6–8 h with 50 ng of virus particles per 5 × 10^4^ cells and with 8 µg/mL polybrene (Sigma Aldrich, St. Louis, MO, USA) in DMEM medium without FCS, penicillin, or streptomycin. Viral transduced cells were selected with 1 µg/mL puromycin (Sigma Aldrich).

### 4.5. Gas6-Conditioned Medium

Gas6-conditioned medium (Gas6-CM) was prepared as described previously [30,31,32,33]. Briefly, Lenti-X 293T-transduced cells were serum-starved in DMEM, supplemented with 1% penicillin/streptomycin, pyruvate (1 mM), and vitamin K (4 µM) (Cheplapharm, Greifswald, Germany) to induce carboxylated Gas6 GLA domain, for 72 h. The supernatant was collected and centrifuged at 1500 rpm for 10 min at 4 °C and filtered (0.22 µm). Gas6 concentrations were evaluated by ELISA, and a standard curve against purified recombinant Gas6 (R&D Systems) was used as a control. The levels of Gas6 were 2 μg/mL.

### 4.6. OAFLS and Synovial Explant Stimulation and Treatments

OAFLS (passages 4–8) and freshly isolated synovial explants were serum-starved overnight. Cells were stimulated with 0.1 ng/mL of recombinant human IL-1β (R&D systems, Oxford, UK), 1 ng/mL of recombinant human TNF-α (R&D systems, Oxford, UK), or 10 ng/mL *Escherichia coli* lipopolysaccharide (LPS) (Invivogen, San Diego, CA, USA) for 24 h. Inhibition of TAM receptor was performed with a pan-TAM inhibitor, RU301 (10 µM) (Axon Medchem, Groningen, The Netherlands), or with a selective Axl inhibitor, RU428 (1 µM) (MedChemExpress, South Brunswick, NJ, USA), 1 h prior to Gas6-CM. Concentrations were determined based on previous studies [33,56]. Gas6-CM was added in a dose-response curve of 16, 8, and 1.6 nM, 1 h prior to LPS stimulation. Supernatants were collected for ELISA or Luminex analyses, and cells were processed for RNA isolation and Western blotting analyses.

### 4.7. qPCR Analysis

Total RNA was extracted using TRI reagent (Sigma, St. Louis, MO, USA) according to the manufacturer’s recommendations. A maximum of 1 µg of mRNA was treated with 1 µL of DNAse (Life Technologies, Carlsbad, CA, USA) for 15 min at room temperature to remove possible genomic DNA, followed by 10 min of inactivation by incubation at 65 °C with 1 µL of 25 mM EDTA (Life Technologies). The cDNA production was performed by using MLV reverse transcriptase (Invitrogen) and oligo(dT) primers. Quantitative polymerase chain reaction (qPCR) analysis was performed with 5 µL of power SYBR green PCR master mix (Applied Biosystems, Waltham, MA, USA), 1 µL of forward primer (2 µM), 1 µL of reverse primer (2 µM), and 3 µL of cDNA. Primer sequences are listed in the Supplementary Table S1. The PCR protocol consisted of 10 min at 95 °C, followed by 40 cycles of 15 s at 95 °C and 60 s at 60 °C. Melting curves were performed to confirm gene-specific amplification. Data were expressed as relative gene expression corrected for the reference gene *GAPDH*, depicted as −ΔC_T_. To determine the fold change in gene expression, log base 2 of relative gene expression corrected for reference gene and unstimulated control (ΔΔCT) was calculated (2^−ΔΔCT^).

### 4.8. Western Blot Analysis

Protein extracts (30–50 µg) produced using RIPA lysis buffer (Cell Signaling) were separated by electrophoresis on bis-acrylamide-SDS PAGE gels and electrically transferred to 0.45 µm pore nitrocellulose membranes (GE Healthcare, Chicago, IL, USA). Non-specific antibody binding was blocked with 5% non-fat dry milk for 1 h. Membranes were incubated with specific primary antibodies against Axl, P-STAT1, P-STAT3 (Cell Signaling), P-Axl (R&D systems), and GAPDH (Sigma Aldrich), and with species-specific immunoglobulins/HRP-conjugated secondary antibodies. Enhanced chemiluminescence ECL prime kit (GE Healthcare) was used to visualize bands using the ImageQuant LAS4000 (Leica).

### 4.9. ELISA

Synovial fluid sAxl (DY154), sMer (DY6488), sTyro3 (DY859), and Gas6 (DY885B) levels were determined using the DuoSet sandwich ELISA kits purchased from R&D Systems (Minneapolis, MN, USA). sAxl and Gas6 in supernatants of OAFLS were also determined. ELISAs were performed according to the manufacturer’s instructions using the DuoSet ELISA Ancillary Reagent Kit 2 (DY008; R&D Systems). Absorbance at 450 nm with a correction wavelength of 540 nm was detected using a microplate reader (CLARIOstar, BMG LABTECH).

### 4.10. Measurement of Cytokines by Multiplex ELISA

The concentrations of IL-1β, CXCL8, IL-6, TNF-α, and CCL2 in synovial fluids and OAFLS supernatants were determined by a Bio-Plex 200 system using a magnetic bead-based multiplex immunoassay (Bio-Rad Laboratories, Hercules, CA, USA). The assay was performed according to manufacturer protocols, using reagents (diluents, calibrators, blocking reagents, and antibody-detecting mixtures) included in the kits. Data analysis was performed with Bio-Plex Manager software (Bio-Rad Laboratories).

### 4.11. Immunohistochemistry

Immunostaining was performed as previously described [16]. OA synovial tissues were used to determine protein expression of Axl, Mer, and Tyro3. Sections of synovial tissues were incubated with rabbit anti-human Axl (1:600; C89E7; Cell Signaling, Danvers, MA, USA), rabbit anti-human Mer (1:2000; ab52968; Abcam, Cambridge, UK), rabbit anti-human Tyro3 (1:500; ab109231; Abcam), or rabbit anti-human IgG (1:74,000; X0936; Agilent Technologies, Santa Clara, CA, USA) overnight at 4 °C and subsequently with biotinylated goat anti-rabbit IgG (1:400; PK-6101; Vector Laboratories, Peterborough, UK) for 30 min at RT. A biotin–streptavidin horseradish peroxidase detection system was used according to the manufacturer’s protocol (PK6101; Vector Laboratories). Bound complexes were visualized with diaminobenzidine by incubation for 10 min at RT. Sections were counterstained with hematoxylin. Pictures were taken with a Leica DMR microscope (Leica Microsystems, Wetzlar, Germany) at 20× and 40× magnification.

### 4.12. Efferocytosis Assay

THP-1 cells were stimulated with PMA (20 ng/mL) for 24 h. Peripheral blood neutrophils were isolated by a density gradient centrifugation method using Ficoll-Paque PLUS (GE Healthcare). Neutrophils were treated with staurosporine (2 µM) (Sigma Aldrich) to induce apoptosis. Apoptotic cells were labeled with pHrodo succinimidyl ester (40 ng/mL) (Life Technologies). Apoptosis was morphologically determined using Giemsa staining (cells exhibited chromatin condensation, nuclear fragmentation, and formation of apoptotic bodies). THP-1 cells were co-incubated with apoptotic cells at a ratio of 1:10. The fluorescence of pHrodo was measured at 590 nm with CLARIOstar. 

### 4.13. Statistical Analysis

The statistical significance between groups was determined by Student’s *t*-tests or one-way analysis of variance (ANOVA) followed by Tukey’s multiple comparison test. Correlations were assessed using Pearson’s correlation coefficient. Data are presented as mean ± SEM; *p* < 0.05 was considered significant. Calculations were performed using the prism 8.0.2 software program for Windows (GraphPad software, San Diego, CA, USA). Outliers were excluded from the analyses.

## Figures and Tables

**Table 1:** Relationship between soluble TAM receptors and Gas6 levels, and inflammatory markers in synovial fluid of OA patients.

	IL-6		CCL2		TNF-α		IL-1β		CXCL8	
	*r*	*p*-Value	*r*	*p*-Value	*r*	*p*-Value	*r*	*p*-Value	*r*	*p*-Value
**Gas6**	−0.401	0.250	0.359	0.252	0.444	0.199	0.093	0.826	−0.314	0.378
**sAxl**	−0.241	0.503	0.266	0.404	0.616	0.058	−0.026	0.952	−0.114	0.753
**sMer**	−0.440	0.204	0.115	0.772	−0.110	0.762	−0.267	0.523	−0.470	0.170
**sTyro3**	−0.245	0.500	−0.485	0.110	−0.369	0.294	−0.613	0.106	−0.007	0.984

Relationship between Gas6, soluble Axl (sAxl), soluble Mer (sMer), soluble Tyro3 (sTyro3), and cytokine levels in synovial fluid of OA patients (*n* = 12). Data are presented as Pearson r value (*r*) and *p*-value for each correlation.

## Data Availability

Data is contained within the article and Supplementary Material.

## Supplementary Materials

The following supporting information can be downloaded at: https://www.mdpi.com/article/10.3390/ph16050703/s1, Figure S1: Correlations between Gas6 and soluble Mer and Tyro3 in synovial fluids of OA patients; Figure S2: Dose response of LPS, IL-1β and TNF-α and the expression of inflammatory markers on OAFLS; Figure S3: Evaluation of efferocytosis mediated by Gas6-conditioned medium in THP-1 cells; Figure S4: Effect of Gas6-CM on cytokine production in the supernatants of OAFLS; Table S1: Primers sequences used in this study.

## References

[1] Loeser R.F., Goldring S.R., Scanzello C.R., Goldring M.B. (2012). Osteoarthritis: A disease of the joint as an organ. Arthritis Rheum..

[2] Liu-Bryan R., Terkeltaub R. (2015). Emerging regulators of the inflammatory process in osteoarthritis. Nat. Rev. Rheumatol..

[3] Sellam J., Berenbaum F. (2010). The role of synovitis in pathophysiology and clinical symptoms of osteoarthritis. Nat. Rev. Rheumatol..

[4] Scanzello C.R., Goldring S.R. (2012). The role of synovitis in osteoarthritis pathogenesis. Bone.

[5] Scanzello C.R., Plaas A., Crow M.K. (2008). Innate immune system activation in osteoarthritis: Is osteoarthritis a chronic wound?. Curr. Opin. Rheumatol..

[6] Scanzello C.R. (2017). Chemokines and inflammation in osteoarthritis: Insights from patients and animal models. J. Orthop. Res..

[7] Van den Bosch M.H.J., van Lent P., van der Kraan P.M. (2020). Identifying effector molecules, cells, and cytokines of innate immunity in OA. Osteoarthr. Cartil..

[8] Maldonado M., Nam J. (2013). The role of changes in extracellular matrix of cartilage in the presence of inflammation on the pathology of osteoarthritis. Biomed. Res. Int..

[9] Lemke G. (2013). Biology of the TAM receptors. Cold Spring Harb. Perspect. Biol..

[10] Lew E.D., Oh J., Burrola P.G., Lax I., Zagorska A., Traves P.G., Schlessinger J., Lemke G. (2014). Differential TAM receptor-ligand-phospholipid interactions delimit differential TAM bioactivities. eLife.

[11] Deng T., Zhang Y., Chen Q., Yan K., Han D. (2012). Toll-like receptor-mediated inhibition of Gas6 and ProS expression facilitates inflammatory cytokine production in mouse macrophages. Immunology.

[12] Rothlin C.V., Ghosh S., Zuniga E.I., Oldstone M.B., Lemke G. (2007). TAM receptors are pleiotropic inhibitors of the innate immune response. Cell.

[13] Zheng S., Hedl M., Abraham C. (2015). TAM receptor-dependent regulation of SOCS3 and MAPKs contributes to proinflammatory cytokine downregulation following chronic NOD2 stimulation of human macrophages. J. Immunol..

[14] Vago J.P., Amaral F.A., van de Loo F.A.J. (2021). Resolving inflammation by TAM receptor activation. Pharmacol. Ther..

[15] Pagani S., Bellan M., Mauro D., Castello L.M., Avanzi G.C., Lewis M.J., Sainaghi P.P., Pitzalis C., Nerviani A. (2020). New Insights into the Role of Tyro3, Axl, and Mer Receptors in Rheumatoid Arthritis. Dis. Markers.

[16] Vullings J., Vago J.P., Waterborg C.E.J., Thurlings R.M., Koenders M.I., van Lent P., van der Kraan P.M., Amaral F.A., van de Loo F.A.J. (2020). Selective Increment of Synovial Soluble TYRO3 Correlates with Disease Severity and Joint Inflammation in Patients with Rheumatoid Arthritis. J. Immunol. Res..

[17] O’Donnell K., Harkes I.C., Dougherty L., Wicks I.P. (1999). Expression of receptor tyrosine kinase Axl and its ligand Gas6 in rheumatoid arthritis: Evidence for a novel endothelial cell survival pathway. Am. J. Pathol..

[18] Bassyouni I.H., El-Wakd M.M., Azab N.A., Bassyouni R.H. (2017). Diminished soluble levels of growth arrest specific protein 6 and tyrosine kinase receptor Axl in patients with rheumatoid arthritis. Int. J. Rheum. Dis..

[19] O’Bryan J.P., Fridell Y.W., Koski R., Varnum B., Liu E.T. (1995). The transforming receptor tyrosine kinase, Axl, is post-translationally regulated by proteolytic cleavage. J. Biol. Chem..

[20] Ekman C., Stenhoff J., Dahlback B. (2010). Gas6 is complexed to the soluble tyrosine kinase receptor Axl in human blood. J. Thromb. Haemost..

[21] Zhenghai S. (2022). Increased serum AXL is associated with radiographic knee osteoarthritis severity. Int. J. Rheum. Dis..

[22] Bhattacharjee M., Balakrishnan L., Renuse S., Advani J., Goel R., Sathe G., Keshava Prasad T.S., Nair B., Jois R., Shankar S. (2016). Synovial fluid proteome in rheumatoid arthritis. Clin. Proteom..

[23] van den Brand B.T., Abdollahi-Roodsaz S., Vermeij E.A., Bennink M.B., Arntz O.J., Rothlin C.V., van den Berg W.B., van de Loo F.A. (2013). Therapeutic efficacy of Tyro3, Axl, and Mer tyrosine kinase agonists in collagen-induced arthritis. Arthritis Rheum..

[24] Waterborg C.E.J., Broeren M.G.A., Blaney Davidson E.N., Koenders M.I., van Lent P., van den Berg W.B., van der Kraan P.M., van de Loo F.A.J. (2019). The level of synovial AXL expression determines the outcome of inflammatory arthritis, possibly depending on the upstream role of TGF-beta1. Rheumatology.

[25] Waterborg C.E.J., Koenders M.I., van Lent P., van der Kraan P.M., van de Loo F.A.J. (2018). Tyro3/Axl/Mertk-deficient mice develop bone marrow edema which is an early pathological marker in rheumatoid arthritis. PLoS ONE.

[26] Waterborg C.E.J., Beermann S., Broeren M.G.A., Bennink M.B., Koenders M.I., van Lent P., van den Berg W.B., van der Kraan P.M., van de Loo F.A.J. (2018). Protective Role of the MER Tyrosine Kinase via Efferocytosis in Rheumatoid Arthritis Models. Front. Immunol..

[27] Thorp E., Vaisar T., Subramanian M., Mautner L., Blobel C., Tabas I. (2011). Shedding of the Mer tyrosine kinase receptor is mediated by ADAM17 protein through a pathway involving reactive oxygen species, protein kinase Cdelta, and p38 mitogen-activated protein kinase (MAPK). J. Biol. Chem..

[28] Rothlin C.V., Carrera-Silva E.A., Bosurgi L., Ghosh S. (2015). TAM receptor signaling in immune homeostasis. Annu. Rev. Immunol..

[29] Feng X., Deng T., Zhang Y., Su S., Wei C., Han D. (2011). Lipopolysaccharide inhibits macrophage phagocytosis of apoptotic neutrophils by regulating the production of tumour necrosis factor alpha and growth arrest-specific gene 6. Immunology.

[30] Happonen K.E., Tran S., Morgelin M., Prince R., Calzavarini S., Angelillo-Scherrer A., Dahlback B. (2016). The Gas6-Axl Protein Interaction Mediates Endothelial Uptake of Platelet Microparticles. J. Biol. Chem..

[31] Geng K., Kumar S., Kimani S.G., Kholodovych V., Kasikara C., Mizuno K., Sandiford O., Rameshwar P., Kotenko S.V., Birge R.B. (2017). Requirement of Gamma-Carboxyglutamic Acid Modification and Phosphatidylserine Binding for the Activation of Tyro3, Axl, and Mertk Receptors by Growth Arrest-Specific 6. Front. Immunol..

[32] Tsou W.I., Nguyen K.Q., Calarese D.A., Garforth S.J., Antes A.L., Smirnov S.V., Almo S.C., Birge R.B., Kotenko S.V. (2014). Receptor tyrosine kinases, TYRO3, AXL, and MER, demonstrate distinct patterns and complex regulation of ligand-induced activation. J. Biol. Chem..

[33] Kimani S.G., Kumar S., Bansal N., Singh K., Kholodovych V., Comollo T., Peng Y., Kotenko S.V., Sarafianos S.G., Bertino J.R. (2017). Small molecule inhibitors block Gas6-inducible TAM activation and tumorigenicity. Sci. Rep..

[34] Jiang L., Chen X.Q., Gao M.J., Lee W., Zhou J., Zhao Y.F., Wang G.D. (2019). The Pros1/Tyro3 axis protects against periodontitis by modulating STAT/SOCS signalling. J. Cell. Mol. Med..

[35] Peng C.K., Wu C.P., Lin J.Y., Peng S.C., Lee C.H., Huang K.L., Shen C.H. (2019). Gas6/Axl signaling attenuates alveolar inflammation in ischemia-reperfusion-induced acute lung injury by up-regulating SOCS3-mediated pathway. PLoS ONE.

[36] Krebs D.L., Hilton D.J. (2001). SOCS proteins: Negative regulators of cytokine signaling. Stem Cells.

[37] Culemann S., Gruneboom A., Nicolas-Avila J.A., Weidner D., Lammle K.F., Rothe T., Quintana J.A., Kirchner P., Krljanac B., Eberhardt M. (2019). Locally renewing resident synovial macrophages provide a protective barrier for the joint. Nature.

[38] Zhang F., Wei K., Slowikowski K., Fonseka C.Y., Rao D.A., Kelly S., Goodman S.M., Tabechian D., Hughes L.B., Salomon-Escoto K. (2019). Defining inflammatory cell states in rheumatoid arthritis joint synovial tissues by integrating single-cell transcriptomics and mass cytometry. Nat. Immunol..

[39] Alivernini S., MacDonald L., Elmesmari A., Finlay S., Tolusso B., Gigante M.R., Petricca L., Di Mario C., Bui L., Perniola S. (2020). Distinct synovial tissue macrophage subsets regulate inflammation and remission in rheumatoid arthritis. Nat. Med..

[40] Sather S., Kenyon K.D., Lefkowitz J.B., Liang X., Varnum B.C., Henson P.M., Graham D.K. (2007). A soluble form of the Mer receptor tyrosine kinase inhibits macrophage clearance of apoptotic cells and platelet aggregation. Blood.

[41] Guo L., Eisenman J.R., Mahimkar R.M., Peschon J.J., Paxton R.J., Black R.A., Johnson R.S. (2002). A proteomic approach for the identification of cell-surface proteins shed by metalloproteases. Mol. Cell. Proteom..

[42] Ekman C., Gottsater A., Lindblad B., Dahlback B. (2010). Plasma concentrations of Gas6 and soluble Axl correlate with disease and predict mortality in patients with critical limb ischemia. Clin. Biochem..

[43] Flem-Karlsen K., Nyakas M., Farstad I.N., McFadden E., Wernhoff P., Jacobsen K.D., Florenes V.A., Maelandsmo G.M. (2020). Soluble AXL as a marker of disease progression and survival in melanoma. PLoS ONE.

[44] Staufer K., Dengler M., Huber H., Marculescu R., Stauber R., Lackner C., Dienes H.P., Kivaranovic D., Schachner C., Zeitlinger M. (2017). The non-invasive serum biomarker soluble Axl accurately detects advanced liver fibrosis and cirrhosis. Cell Death Dis..

[45] Dengler M., Staufer K., Huber H., Stauber R., Bantel H., Weiss K.H., Starlinger P., Pock H., Kloters-Plachky P., Gotthardt D.N. (2017). Soluble Axl is an accurate biomarker of cirrhosis and hepatocellular carcinoma development: Results from a large scale multicenter analysis. Oncotarget.

[46] Gui S., Zhou S., Liu M., Zhang Y., Gao L., Wang T., Zhou R. (2021). Elevated Levels of Soluble Axl (sAxl) Regulates Key Angiogenic Molecules to Induce Placental Endothelial Dysfunction and a Preeclampsia-Like Phenotype. Front. Physiol..

[47] Weinger J.G., Omari K.M., Marsden K., Raine C.S., Shafit-Zagardo B. (2009). Up-regulation of soluble Axl and Mer receptor tyrosine kinases negatively correlates with Gas6 in established multiple sclerosis lesions. Am. J. Pathol..

[48] Ruiz-Heiland G., Zhao Y., Derer A., Braun T., Engelke K., Neumann E., Mueller-Ladner U., Liu Y., Zwerina J., Schett G. (2014). Deletion of the receptor tyrosine kinase Tyro3 inhibits synovial hyperplasia and bone damage in arthritis. Ann. Rheum Dis.

[49] Kapoor M., Martel-Pelletier J., Lajeunesse D., Pelletier J.P., Fahmi H. (2011). Role of proinflammatory cytokines in the pathophysiology of osteoarthritis. Nat. Rev. Rheumatol..

[50] Goldring M.B., Otero M., Tsuchimochi K., Ijiri K., Li Y. (2008). Defining the roles of inflammatory and anabolic cytokines in cartilage metabolism. Ann. Rheum. Dis..

[51] Kraus V.B., McDaniel G., Huebner J.L., Stabler T.V., Pieper C.F., Shipes S.W., Petry N.A., Low P.S., Shen J., McNearney T.A. (2016). Direct in vivo evidence of activated macrophages in human osteoarthritis. Osteoarthr. Cartil..

[52] Guo Q., Liu Z., Wang M., Guo S., Cong H., Liu L. (2021). Analysis on the expression and value of CCL2 and CCL3 in patients with osteoarthritis. Exp. Mol. Pathol..

[53] Fleischmann R.M., Bliddal H., Blanco F.J., Schnitzer T.J., Peterfy C., Chen S., Wang L., Feng S., Conaghan P.G., Berenbaum F. (2019). A Phase II Trial of Lutikizumab, an Anti-Interleukin-1alpha/beta Dual Variable Domain Immunoglobulin, in Knee Osteoarthritis Patients with Synovitis. Arthritis Rheumatol..

[54] Maksymowych W.P., Russell A.S., Chiu P., Yan A., Jones N., Clare T., Lambert R.G. (2012). Targeting tumour necrosis factor alleviates signs and symptoms of inflammatory osteoarthritis of the knee. Arthritis Res. Ther..

[55] Chevalier X., Goupille P., Beaulieu A.D., Burch F.X., Bensen W.G., Conrozier T., Loeuille D., Kivitz A.J., Silver D., Appleton B.E. (2009). Intraarticular injection of anakinra in osteoarthritis of the knee: A multicenter, randomized, double-blind, placebo-controlled study. Arthritis Rheum..

[56] Holland S.J., Pan A., Franci C., Hu Y., Chang B., Li W., Duan M., Torneros A., Yu J., Heckrodt T.J. (2010). R428, a selective small molecule inhibitor of Axl kinase, blocks tumor spread and prolongs survival in models of metastatic breast cancer. Cancer Res..

