# Supplementary material for: Gas6/Axl Axis Activation Dampens the Inflammatory Response in Osteoarthritic Fibroblast-like Synoviocytes and Synovial Explants
Source: Pharmaceuticals (Basel). 2023 May 6;16(5):703. doi: 10.3390/ph16050703 (PMC10222253; doi:10.3390/ph16050703)
Supplement: Supplementary file 1 [file pharmaceuticals-16-00703-s001.zip › pharmaceuticals-2340959-supplementary.pdf]

Supplementary figure 1

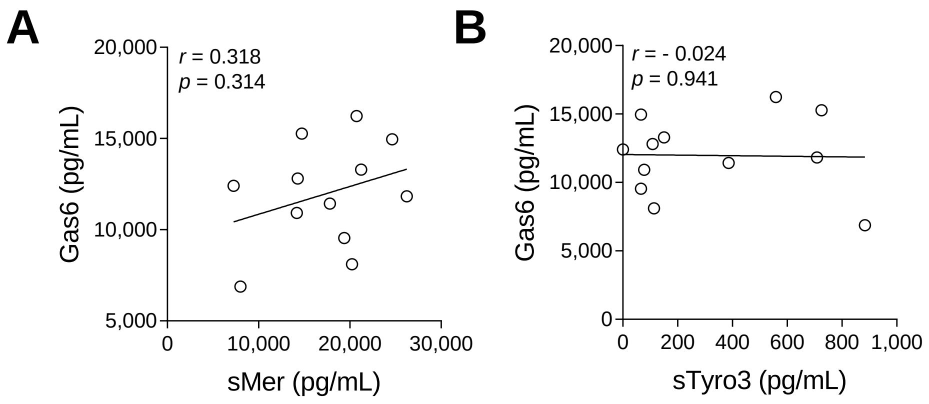

Supplementary figure 1. Correlations between Gas6 and soluble Mer and Tyro3 in synovial fluids of OA patients. Soluble Mer (sMer), soluble Tyro3 (sTyro3) and Gas6 levels were determined in synovial fluids of OA patients (*n*=12) by ELISA. The correlations between sMer and Gas6 (A) and sTyro3 and Gas6 (B) were evaluated by Person's coefficients. Data are presented as the Pearson r value (r) and p value (*p*) for each correlation.

Supplementary figure 2

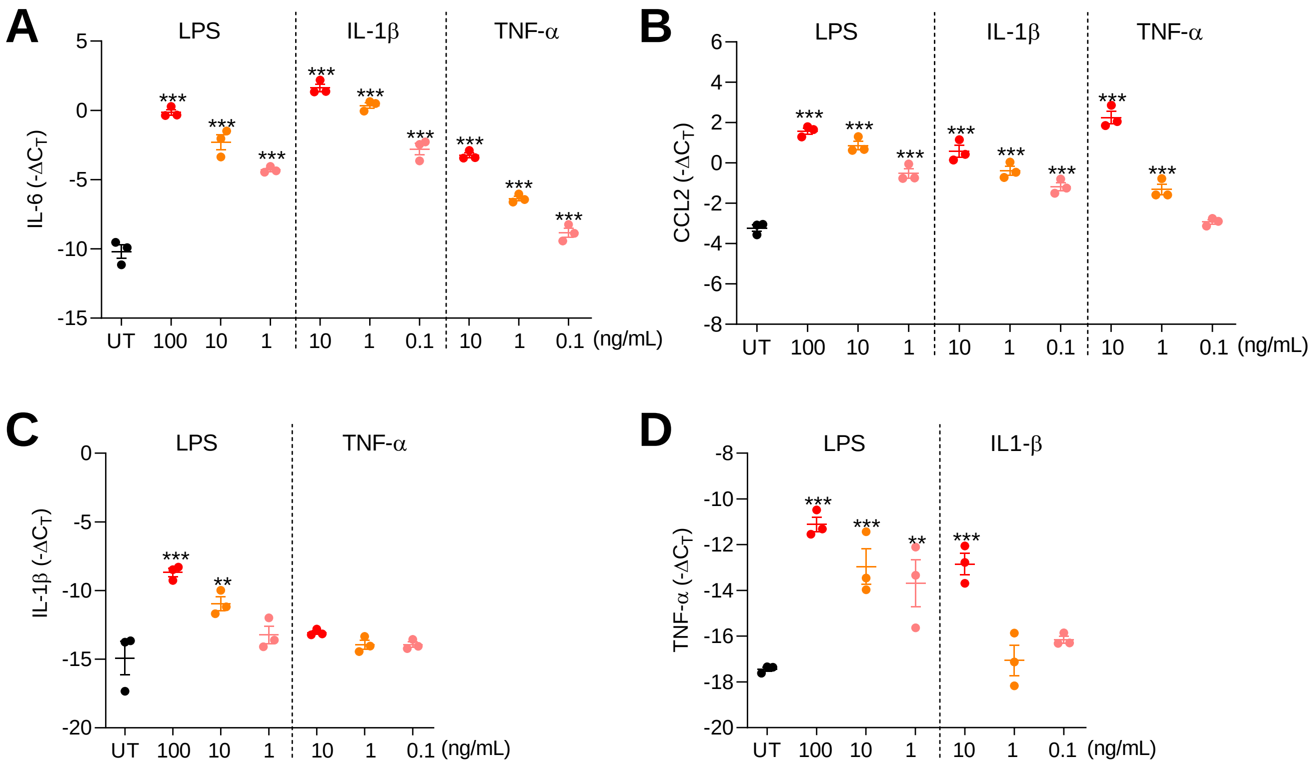

Supplementary figure 2. Dose response of LPS, IL-1β and TNF-α and the expression of inflammatory markers on OAFLS. OA synovial fibroblasts (OAFLS) were stimulated with LPS (10ng/ml) or the recombinants human IL-1β (0.1ng/ml) and TNF-α (1ng/ml) for 24h. Cells were processed for qPCR analyses of the pro-inflammatory cytokines IL-6 (A), CCL2 (B), IL-1β (C) and TNF-α (D). Results are shown as the mean ± SEM. $*P < 0.05$, $***P < 0.001$ when comparing LPS with untreated (UT) group. P values were determined by ANOVA with post hoc Tukey's multiple comparison test.

Supplementary figure 3

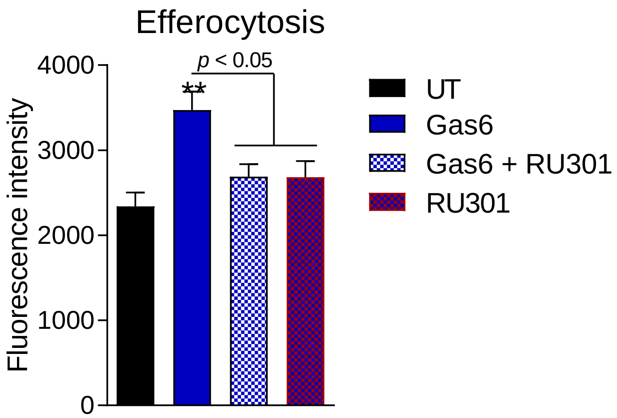

Supplementary figure 3. Evaluation of efferocytosis mediated by Gas6-conditioned medium in THP-1 cells. THP-1 cells were stimulated with PMA (20 ng/ml) for 24h. Human peripheral blood neutrophils were pre-treated with staurosporine (2 µM) to induce apoptosis and labelled with pHrodo succinimidyl ester (40ng/ml). THP-1 cells were pre-treated with a pan-TAM inhibitor RU301 (10uM). Apoptotic neutrophils were pre-incubated with Gas6-CM (16µm) for 30 minutes. The efferocytosis assay was performed by co-culturing THP-1 cells with apoptotic neutrophils in a proportion of 10 neutrophils per macrophage. Fluorescence of pHrodo was measured at 590 nm. $^*P < 0.05$, when comparing Gas6-CM treated group with untreated (UT) group. P values were determined by ANOVA with post hoc Tukey's multiple comparison test.

Supplementary figure 4

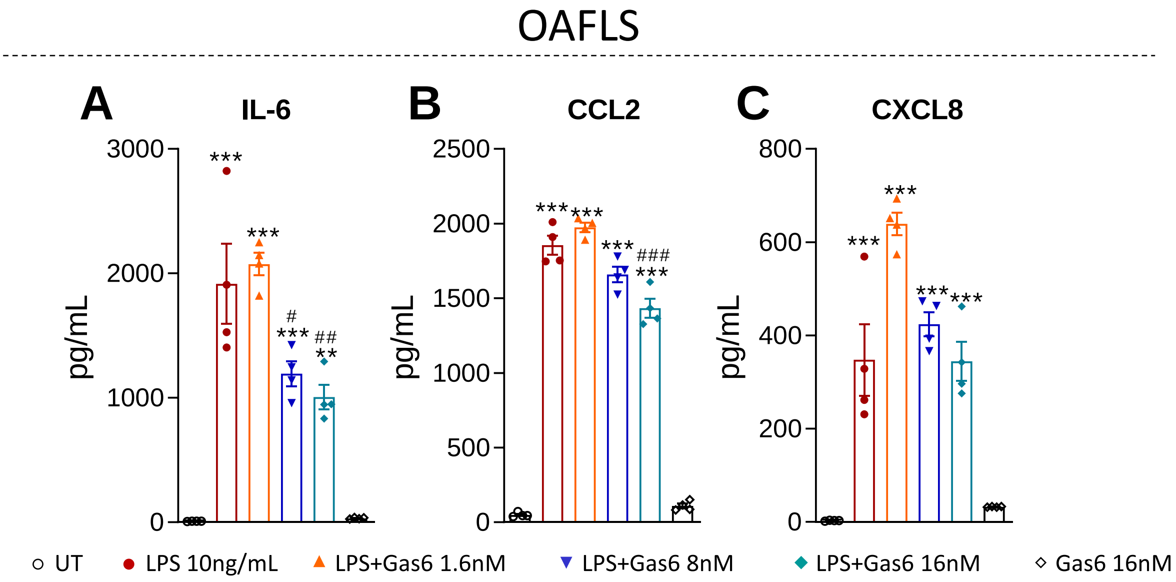

Supplementary figure 4. Effect of Gas6-CM on cytokine production in the supernatants of OAFLS. OA synovial fibroblasts (OAFLS) were pre-treated with Gas6-CM (16nM) for 1h and then, stimulated for 24h with LPS (10ng/ml). The pro-inflammatory cytokines IL-6 (A), CCL2 (B), and CXCL8 (C) were analyzed by multiplex ELISA in the supernatants. Results are shown as the mean ± SEM. ***P < 0.001 when comparing with untreated (UT) group. $^{\#}P < 0.05$, $^{\#\#}P < 0.01$, and $^{\#\#\#}P < 0.001$ when comparing LPS+Gas6 treated with LPS group. P values were determined by ANOVA with post hoc Tukey's test (multiple groups).

Supplementary Table 1

Table S1. Primers sequences used in this study.

Gene	Forward sequence	Reverse sequence
IL6	AGCCCACCGGGAACGA	GGACCGAAGGCGCTTGT
CCL2	CGCTCAGCCAGATGCAATC	GCCTCTGCACTGAGATCTTCCT
TNFA	TCTTCTCGAACCCCGAGTGA	CCTCTGATGGCACCACCAG
IL1B	TGGGTAATTTTTGGGATCTACACTCT	AATCTGTACCTGTCCTGCGTGTT
CXCL8	AGAAGTTTTTGAAGAGGGCTGAGA	CAGACCCACACAATACATGAAGTG
AXL	CAGCTCAGAATCACCTCCC	ACACGAAGGTCTGATGTCC
MER	TCTGGGTGAAGGAGAGTTTGG	TCCCGCTGTGAAGAGTTGTC
TYRO3	CCACTGTTCACCTTCAAGCA	CCTGTGTCACCTGAACTGTAC
GAS6	ACTTCTTCTGCCTGTGTAAAGC	TCCTGGCTGCATTCGTTGAC
GAPDH	ATCTTCTTTTGCGTCGCCAG	TTCCCCATGGTGTCTGAGC
